# Reconstruction of Misalignment Aberrations for Cylindrical Surfaces with Complex Parameters in Pseudo Lateral Shearing Interferometry

**DOI:** 10.3390/s26061854

**Published:** 2026-03-15

**Authors:** Yuxuan Ren, Weizhou Luo, Yang Chen, Le Zhao, Liuqing He, Siqi Zhang, Kuo Hai, Xiaodong Zhang, Zhongming Zang

**Affiliations:** 1 State Key Laboratory of Precision Measuring Technology & Instruments, Laboratory of Micro/Nano Manufacturing Technology, Tianjin University, Tianjin 300072, China; 2023202055@tju.edu.cn; 2 Sichuan Precision and Ultra-Precision Machining Engineering Technology Center, Chengdu 610200, China; luowz599@gmail.com (W.L.); chenyanggoku@163.com (Y.C.); lzhao@cjlu.edu.cn (L.Z.); heliuqing95@163.com (L.H.); m17801003047@163.com (S.Z.); haikuo_6s@126.com (K.H.)

**Keywords:** non-null testing, reconstruction algorithm in one direction, misalignment aberrations, two-dimensional pseudo lateral shearing interferometry, optical detection technology

## Abstract

**Highlights:**

**What are the main findings?**

- A novel non-null interferometric measurement method for cylindrical surfaces with complex parameters is presented.
- Misalignment aberrations can be reconstructed using only the partial derivative in one direction.

**What are the implications of the main findings?**

- Various wavefront errors and the impact of other optics are eliminated in measurements.
- The reconstruction of misalignment aberrations does not require complex matrix operations or model matching.

**Abstract:**

Cylindrical surfaces with complex parameters (CSCPs) have off-axis and aspheric properties. High-precision measurement of cylindrical surfaces is a key research focus in optical metrology. Two-dimensional pseudo lateral shearing interferometry (2DPLSI) enables non-null generalized interferometry for cylindrical surfaces. However, due to the non-rotational symmetry of cylindrical surfaces with complex parameters, measuring them using two-dimensional pseudo lateral shearing interferometry inevitably introduces misalignment aberrations, degrading the accuracy of cylindrical surface reconstruction. To address this issue, we propose a novel non-null testing method: the cylindrical surface is translated in the orthogonal directions to carry out the shearing process, and wavefront errors are eliminated through second-order differencing. Furthermore, a reconstruction algorithm in one direction is proposed. Using only the partial derivative in the x direction, the wavefront error of misalignment aberrations can be reconstructed, enabling high-precision recovery of the cylindrical surface. Experimental results using a Fizeau interferometer demonstrate that the proposed method effectively reconstructs misalignment aberrations. The reconstructed cylindrical surface achieves a peak-to-valley (PV) value of 0.45λ (λ = 632.8 nm) and a root-mean-square (RMS) value of 0.12λ, comparable to the 0.37λ PV and 0.09λ RMS obtained via null testing. The repeatability of the proposed method is superior to λ/1000 RMS.

## 1. Introduction

With the development of optical manufacturing technologies, the measurement accuracy of aspheric optics has also been significantly improved. Cylindrical optics have curvature only in a single direction and no curvature in the orthogonal direction. Owing to this distinctive geometric characteristic, the manufacturing and measurement of cylindrical optics remain challenging. Cylindrical optics are widely used in line focusing or aberration correction [1] and are commonly applied in fields such as holographic illumination [2], medical diagnostics [3], beam shaping [4], and optical microfabrication [5]. As key components in high-precision instruments, cylindrical optics require high-precision surface quality. Therefore, higher demands are placed on the manufacturing and measurement technologies of cylindrical optics. The manufacturing quality relies heavily on the metrology precision, making high-accuracy surface measurement indispensable.

There are various measurement methods for cylindrical optics. The contact detection method of cylindrical optics suffers from low efficiency and limited accuracy and may damage the surface of the element, such as in coordinate-measuring machines [6]. Non-contact optical measurement techniques include the Shack–Hartmann wavefront sensor [7,8,9], curvature sensing and Luphoscan. To achieve high-precision measurement, the Shack–Hartmann wavefront sensor requires a microlens array with high fabrication accuracy, which increases the difficulty and cost of manufacturing. Curvature sensing has been widely applied in adaptive optics and astronomical imaging [10]. Curvature sensors have a relatively small dynamic measurement range [11,12]. When they are applied to the measurement of large-aperture cylindrical optics with complex parameters, the required algorithms become more complex, and the wavefront reconstruction process becomes more challenging. Luphoscan faces challenges in maintaining real-time alignment between the probe and the surface normal of cylindrical optics, which can compromise measurement accuracy. Consequently, interferometry [13] has become the primary method for measuring cylindrical optics due to its high precision. Interferometric measurement methods can be broadly classified into null and non-null testing. Null testing includes the stigmatic null testing method [14,15], the null-compensating lens method [16,17], and the computer-generated hologram (CGH) [18,19,20,21,22]. Although null testing achieves high-precision measurements with the help of null compensators, it lacks universality. In contrast, non-null testing [23,24] imposes lower requirements on the compensating lens (CL), primarily relying on algorithms to compensate for errors caused by insufficient accuracy of the compensating lens. It offers higher universality but may introduce additional errors.

In non-null testing, interference fringes contain multiple errors and cannot directly reflect the test surface figure. It is necessary to eliminate errors and reconstruct the true surface figure using appropriate methods. Shi et al. [25] identified retrace errors as one of the dominant error sources in non-null testing and proposed several compensation approaches. In addition, misalignment aberrations [26,27] are also major factors that degrade measurement accuracy. Chen et al. [28,29] analyzed the misalignment aberrations introduced by different spatial orientations of cylindrical surfaces in non-null testing. They established a mathematical model of misalignment aberrations by deriving the optical path difference and proposed a corresponding separation method, enabling effective separation of the misalignment aberrations. Peng et al. [30] employed ordinary least squares to fit and eliminate low-order misalignment aberrations in cylindrical surface measurements and then used Legendre polynomials to separate high-order misalignment aberrations. They concluded that the coefficients of the low-order aberrations are linearly related to those of the high-order aberrations. However, existing methods for misalignment aberrations removal mainly focus on spherical cylinders, and related research on CSCPs remains limited. Chen et al. [31,32] proposed the two-dimensional pseudo lateral shearing interferometry, which successfully eliminates various wavefront errors. They also simulated the effects of system errors and random errors on the measurement accuracy of two-dimensional pseudo lateral shearing interferometry, determining the optimal measurement system parameters for CSCPs. Compared with null testing methods, two-dimensional pseudo lateral shearing interferometry offers higher universality and lower cost. Compared with other non-null testing methods, two-dimensional pseudo lateral shearing interferometry can effectively eliminate the reflection wavefront error of the compensating lens and the retrace error. Consequently, the use of two-dimensional pseudo lateral shearing interferometry for CSCP measurement offers significant advantages. However, when measuring CSCPs using two-dimensional pseudo lateral shearing interferometry, misalignment aberrations are also difficult to completely eliminate, thereby affecting the measurement accuracy. Therefore, this paper fills that research gap.

This paper develops a novel non-null interferometric method with a particular focus on addressing misalignment aberrations in the measurement of CSCPs. We first eliminate various wavefront errors using the pseudo lateral shearing interferometry method. Then, by translating the CSCP conjugately along the x direction, misalignment aberrations are eliminated through the second-order differential wavefront. A reconstruction algorithm in one direction is subsequently proposed to obtain first-order differential wavefronts that are free of misalignment aberrations, enabling accurate reconstruction of the misalignment aberrations. The approach directly processes the measurement data and avoids complex matrix operations or model matching. Finally, the cylindrical surface figure is reconstructed from the first-order differential wavefronts in the orthogonal directions, resulting in improved accuracy in non-null testing of CSCPs. Section 2 introduces the interferometric system for CSCPs and presents a novel measurement method. Section 3 presents the reconstruction algorithm in one direction and verifies its feasibility through simulation. Section 4 experimentally validates the measurement accuracy of the proposed method. Section 5 discusses the results. Section 6 concludes the paper.

## 2. Non-Null Interferometric System and Measurement Method

The CSCP is designed as an off-axis aspheric concave cylindrical surface, and the compensating lens is a spherical convex cylindrical surface. By appropriately selecting the beam reduction ratio and relative positions, the two together form a collimating and beam-reducing system. For commonly used CSCPs, the applicable parameter range of the curvature radius is typically above 400 mm; a smaller curvature radius would substantially increase the complexity of the compensating lens design. In addition, the shear distance is mainly determined by the shear step and spatial resolution of the interferometer.

**Figure 1:**
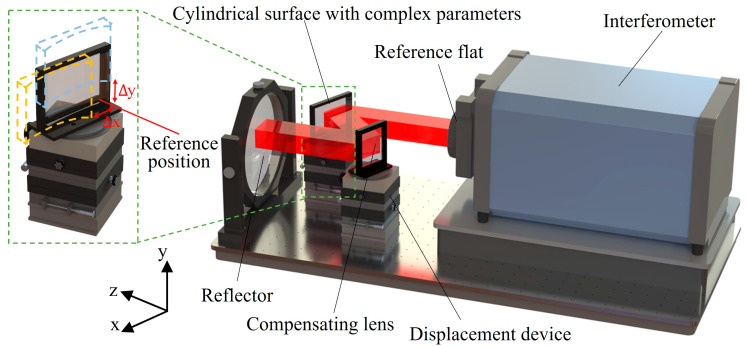
Schematic of the non-null interferometric system.

The non-null interferometric system for CSCPs is shown in Figure 1, which consists of a Fizeau interferometer, the CSCP, the compensating lens, and a plane reflector. The interferometer emits collimated light, part of which is reflected by the reference flat as reference light, while the remaining portion passes through and is reflected by the CSCP and is subsequently reflected by the compensating lens, resulting in a collimated outgoing light. The collimated light is then reflected by a plane reflector and travels back approximately along its original path to the interferometer, forming the test light. The reference light interferes with the test light, and the measurement result obtained from the interferometer contains the reflection wavefront error of the CSCP. By translating the CSCP multiple times along orthogonal directions using displacement devices, measurement results at different spatial positions can be obtained.

The measurement result also contains various wavefront errors, such as a system error and retrace error, which are eliminated using the two-dimensional pseudo lateral shearing interferometry. Two-dimensional pseudo lateral shearing interferometry differs from conventional shearing interferometry. Two-dimensional pseudo lateral shearing interferometry realizes the shearing process by translating the CSCP in orthogonal directions [31]. As shown in Figure 1, when the CSCP is positioned at the reference position, the first measurement result can be obtained in the interferometer. After translating the CSCP a tiny distance along the y direction, the second measurement result can be obtained in the interferometer. The shearing is performed by subtracting the measurement results before and after the displacement. The shearing process for the translation of the CSCP along the x direction is similar. A detailed analysis of the optical train that performs the shearing, as well as the method for eliminating the wavefront errors, is presented below.

The measurement result *W*(*x*, *y*) obtained from the interferometer includes the reflection wavefront error of the CSCP *W*_CSCP_(*x*, *y*), the system error *W*_SE_(*x*, *y*), the reflection wavefront error of the compensating lens *W*_CL_(*x*, *y*), as well as the retrace error *W*_RE_(*x*, *y*). Thus, we can write:

(1) $$W\left(x,y\right)=W_{\mathrm{CSC}P}\left(x,y\right)+W_{\mathrm{SE}}\left(x,y\right)+W_{\mathrm{CL}}\left(x,y\right)+W_{\mathrm{RE}}\left(x,y\right).$$

Only the CSCP is translated by a tiny distance Δ*x* or Δ*y* along the x or y direction, while the other optical components remain stationary. Translating the CSCP along the non-curvature direction (y direction) does not introduce misalignment aberrations. However, when the CSCP is translated along the curvature direction (x direction), misalignment aberrations are inevitably introduced due to its non-rotational symmetry. Consequently, the measurement result contains not only low-order misalignment aberrations but also higher-order misalignment aberrations, such as x-coma 1/2(5*x*^3^ − 3*x*). The superposition of misalignment aberrations and *W*_CSCP_(*x*, *y*) leads to inaccuracies in reconstructing the cylindrical surface figure from the two differential wavefronts obtained along the orthogonal directions. Thus, the measurement results obtained using the two-dimensional pseudo lateral shearing interferometry method can be written as follows [31]:

(2) $$\begin{array}{l} W_{1}\left(x,y\right)=W_{\mathrm{CSC}P}\left(x,y\right)+W_{\mathrm{SE}}\left(x,y\right)+W_{\mathrm{CL}}\left(x,y\right)+W_{\mathrm{RE}}\left(x,y\right)\\ W_{2}\left(x,y\right)=W_{\mathrm{CSC}P}\left(x+\Delta x,y\right)+W_{\mathrm{SE}}\left(x,y\right)+W_{\mathrm{CL}}\left(x,y\right)+W_{\mathrm{RE}}\left(x,y\right)+\phi_{MAx}\\ W_{3}\left(x,y\right)=W_{\mathrm{CSC}P}\left(x+\Delta x,y+\Delta y\right)+W_{\mathrm{SE}}\left(x,y\right)+W_{\mathrm{CL}}\left(x,y\right)+W_{\mathrm{RE}}\left(x,y\right)+\phi_{MAx} \end{array},$$

where Δ*x* and Δ*y* denote tiny distances, specifically corresponding to the size of one CCD pixel in the Fizeau interferometer. $\phi_{MAx}$ denotes the misalignment aberrations introduced by translating along the x direction.

As shown in Equation (2), even when a sufficiently small displacement Δ*x* is used, the existing displacement process of the CSCP in two-dimensional pseudo lateral shearing interferometry cannot effectively eliminate $\phi_{MAx}$ through the first-order differential wavefront *W*_2_(*x*, *y*) − *W*_1_(*x*, *y*). Based on this, a novel measurement method is proposed, in which $\phi_{MAx}$ and various wavefront errors are eliminated by introducing an additional translation of the CSCP in two-dimensional pseudo lateral shearing interferometry.

**Figure 2:**
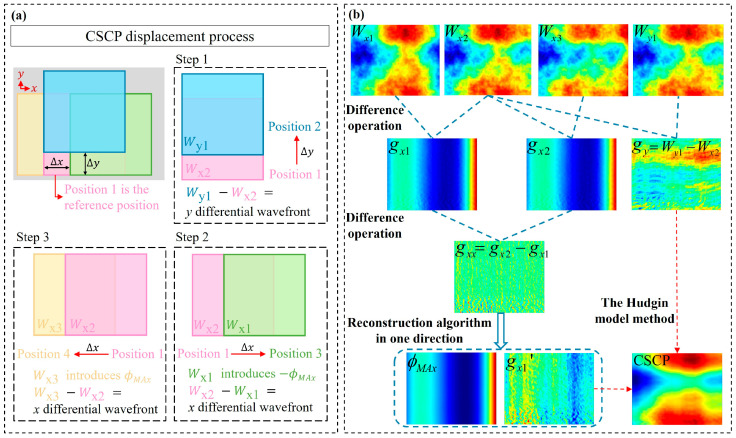
Pseudo-shear measurement procedure. (**a**) CSCP displacement process; (**b**) algorithm flow.

The measurement procedure of the proposed method is illustrated in Figure 2. During the measurement, the relative position between the compensating lens and the CSCP is adjusted to render the interference fringes as sparse as possible, thereby obtaining the measurement result *W_x_*_2_(*x*, *y*) at the reference position. Based on the reference position, a translation of the CSCP along the y direction yields *W_y_*_1_(*x*, *y*). The CSCP is subsequently translated back to the reference position, and conjugate translations along the x direction are performed to obtain *W_x_*_1_(*x*, *y*) and *W_x_*_3_(*x*, *y*). Conjugate translations can be defined as the translations of the CSCP from the reference position along the positive and negative directions of the x-axis, respectively. Due to the tiny shear distance, it is assumed that the same $\phi_{MAx}$ is introduced during the conjugate translations along the x direction. The results of the four measurements are given in Equation (3).

(3) $$\begin{array}{l} W_{x1}\left(x,y\right)=W_{\mathrm{CSC}P}\left(x-\Delta x,y\right)+W_{\mathrm{SE}}\left(x,y\right)+W_{\mathrm{CL}}\left(x,y\right)+W_{\mathrm{RE}}\left(x,y\right)-\phi_{MAx}\\ \begin{array}{l} W_{x2}\left(x,y\right)=W_{\mathrm{CSC}P}\left(x,y\right)+W_{\mathrm{SE}}\left(x,y\right)+W_{\mathrm{CL}}\left(x,y\right)+W_{\mathrm{RE}}\left(x,y\right)\\ W_{x3}\left(x,y\right)=W_{\mathrm{CSC}P}\left(x+\Delta x,y\right)+W_{\mathrm{SE}}\left(x,y\right)+W_{\mathrm{CL}}\left(x,y\right)+W_{\mathrm{RE}}\left(x,y\right)+\phi_{MAx}\\ W_{y1}\left(x,y\right)=W_{\mathrm{CSC}P}\left(x,y+\Delta y\right)+W_{\mathrm{SE}}\left(x,y\right)+W_{\mathrm{CL}}\left(x,y\right)+W_{\mathrm{RE}}\left(x,y\right) \end{array} \end{array}.$$

During translations of the CSCP along orthogonal directions, the position of the beam on the other optical elements besides the CSCP remains unchanged. Therefore, all wavefront errors except for the *W*_CSCP_(*x*, *y*) can be effectively eliminated by subtracting the measurement results, thereby achieving the pseudo-shearing effect. The first-order y-differential wavefront obtained by translating the CSCP along the y direction is given in Equation (4).

(4) $$g_{y}(x,y)=W_{y1}\left(x,y\right)-W_{x2}\left(x,y\right)=W_{\mathrm{CSC}P}\left(x,y+\Delta y\right)-W_{\mathrm{CSC}P}\left(x,y\right),$$

where *g_y_*(*x*, *y*) denotes the first-order y-differential wavefront.

When the CSCP is translated along the x direction, $\phi_{MAx}$ can be effectively eliminated through a second-order difference in the measurement results, as shown in Equation (5).

(5) $$\begin{array}{ll} g_{x1}(x,y) & =W_{x2}(x,y)-W_{x1}(x,y)=W_{CSCP}(x,y)-W_{CSCP}(x-\Delta x,y)+\phi_{MAx}\\ g_{x2}(x,y) & =W_{x3}(x,y)-W_{x2}(x,y)=W_{CSCP}(x+\Delta x,y)-W_{CSCP}(x,y)+\phi_{MAx}\\ g_{xx}(x,y) & =g_{x2}(x,y)-g_{x1}(x,y)\\ & =W_{CSCP}(x+\Delta x,y)+W_{CSCP}(x-\Delta x,y)-2W_{CSCP}(x,y) \end{array},$$

where *g_x_*_1_(*x*, *y*) and *g_x_*_2_(*x*, *y*) denote the first-order x-differential wavefronts. *g_xx_*(*x*, *y*) denotes the second-order x-differential wavefront.

The differential wavefronts can be approximated as the gradients of the wavefront when Δ*x* is sufficiently small [31]. According to Equation (5), only *g_xx_*(*x*, *y*) is free of $\phi_{MAx}$. However, *W*_CSCP_(*x*, *y*) cannot be directly calculated based on *g_xx_*(*x*, *y*). Therefore, the reconstruction algorithm in one direction is required to obtain $\phi_{MAx}$, and the Hudgin model method [33,34,35] is then employed to achieve high-precision surface reconstruction of the CSCP using the first-order differential wavefronts free of $\phi_{MAx}$ in the orthogonal directions.

## 3. Reconstruction Algorithm in One Direction

This section primarily introduces a method based on time–frequency domain transformation for reconstructing $\phi_{MAx}$ in one direction. It is worth noting that shear interference causes loss of original wavefront information, and noise is amplified during integration, both of which contribute to errors in wavefront reconstruction. To address these issues, wavefront prolongation and noise suppression methods are introduced to optimize the reconstruction process, thereby improving the reconstruction accuracy.

### 3.1. Principle of Reconstruction in One Direction

The reconstruction of $\phi_{MAx}$ from *g_xx_*(*x*, *y*) in one direction usually requires complex algorithms. An extended form of the Wiener deconvolution [36] has been used in our work to perform this reconstruction.

Cylindrical optical components only have curvature in a single direction (x direction). Misalignment aberrations are introduced only when the CSCP is translated along the x direction, whereas translation along the y direction does not introduce misalignment aberrations. Consequently, the significance of reconstruction in one direction lies in the fact that $\phi_{MAx}$ can be reconstructed using only the partial derivative in the x direction. The mathematical relationship between the frequency domain and differential wavefronts is established through Fourier transformation. In Equation (5), substituting *x* with *x* + Δ*x* in *g_x_*_1_(*x*, *y*) yields *g_x_*_1_(*x* + Δ*x*, *y*). It can be derived that *g_x_*_1_(*x* + Δ*x*, *y*) is equal to *g_x_*_2_(*x*, *y*); therefore, Equation (6) can be obtained. The Fourier transform of Equation (6) is performed to obtain *W*(*u*, *v*), thereby converting the spatial domain into the frequency domain. Because *g_xx_*(*x*, *y*) does not contain the $\phi_{MAx}$, the Fourier transform of *g_xx_*(*x*, *y*) yields *W*(*u*, *v*) in Equation (7), which is also free of the $\phi_{MAx}$. A translation by an amount Δx in real space corresponds to multiplication by $e^{i2\pi u\Delta x}$ in the Fourier space. Thus, we can write:

(6) $$g_{xx}=g_{x2}\left(x,y\right)-g_{x1}\left(x,y\right)=g_{x1}\left(x+\Delta x,y\right)-g_{x1}\left(x,y\right)$$

and

(7) $$W\left(u,v\right)=F\left\{g_{xx}\left(x,y\right)\right\}=G(u+\Delta x,v)-G(u,v)=(e^{i2\pi u\Delta x}-1)G(u,v),$$

where *u* and *v* are the frequency coordinates in the 2D Fourier space. *W*(*u*, *v*) and *G*(*u*, *v*) are the Fourier transforms of *g_xx_*(*x*, *y*) and *g_x_*_1_(*x*, *y*) respectively.

According to Equation (7), *g_ref_*(*x*, *y*) can be further obtained by taking an inverse Fourier transform of *G*(*u*, *v*). Thus, we can write:

(8) $$G(u,v)=\frac{W(u,v)}{e^{i2\pi u\Delta x}-1}=\frac{W(u,v)}{A(u)}=\frac{A{\left(u\right)}^{*}}{{\left|A(u)\right|}^{2}}W(u,v)$$

and

(9) $$g_{ref}\left(x,y\right)=F{\left\{G(u,v)\right\}}^{-1}=W_{\mathrm{CSC}P}\left(x,y\right)-W_{\mathrm{CSC}P}\left(x-\Delta x,y\right),$$

where A(u) = $e^{i2\pi u\Delta x}-1$.

Since translating the CSCP along the x direction primarily introduces x misalignment aberrations, $\phi_{MAx}$ is obtained by fitting *g_x_*_1_(*x*, *y*) − *g_ref_*(*x*, *y*) using 2D Legendre polynomials along the x direction. The first-order differential wavefront *g_x_*_1_(*x*, *y*)′ that is free of $\phi_{MAx}$ can be calculated using Equation (10).

(10) $$g_{x1}{\left(x,y\right)}^{\prime }=g_{x1}\left(x,y\right)-\phi_{MAx}.$$

### 3.2. Wavefront Prolongation Method

Compared with conventional shear interferometry, the proposed method realizes the shearing process by translating the CSCP. This process does not occur in physical space but is implemented during data processing. Shearing interference inevitably loses part of the original wavefront information, and the proposed algorithm considers information variations only in one direction. Consequently, boundary artifacts are likely to occur during wavefront reconstruction, leading to degraded reconstruction quality. Wavefront prolongation can be used to compensate for information loss and ensure the integrity of the reconstructed wavefront.

Assuming the original wavefront distribution function *W*(*x*) is defined on the interval [0, *a*], a periodic function *W_t_*(*x*) with period 2*a* can be constructed via even prolongation [37]. *W_t_*(*x*) is an axisymmetric function, symmetric about *x* = a:

(11) $$W_{t}\left(x\right)=\left\{\begin{array}{c} W\left(x\right),x\in [0,\;a]\\ W\left(2a-x\right),x\in (a,\;2a] \end{array}\right..$$

Shearing is performed on Equation (11), yielding the following differential function:

(12) $$g_{a}(x)=W_{t}\left(x\right)-W_{t}\left(x-\Delta x\right),$$

where *g_a_*(*x*) is periodic with period 2*a* and odd in the variable *x*.

To ensure the integrity of the wavefront information, Equation (12) must be converted into a full-rank system of algebraic equations, which requires at least *N* sampling points of *g_a_*(*x*). *g_a_*(*x*) and *W_t_*(*x*) are discretely sampled on the interval [0, 2*a*], i.e., $G_{a}=(g_{0}^{a},\;\ldots ,\;g_{N-1}^{a})$, $W_{a}=(W_{0}^{a},\;\ldots ,\;W_{N-1}^{a})$, thus establishing a system of linear equations:

(13) $$A_{a}W_{a}=G_{a},$$

where *A_a_* denotes the periodic prolongation shearing matrix. If $|A_{a}|\neq 0$, the discrete wavefront matrix W*_a_* can be directly obtained.

**Figure 3:**
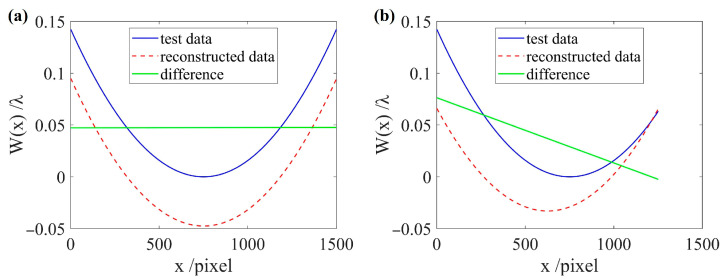
Comparison of reconstruction results for different functions. (**a**) The test data is an axisymmetric function; (**b**) the test data is a non-axisymmetric function.

The feasibility of the wavefront prolongation method is verified using one-dimensional simulation data. When the test data is an axisymmetric function and a non-axisymmetric function, respectively, the reconstruction results obtained by using Equations (6)–(9) are shown in Figure 3.

In Figure 3, the blue solid line represents the known test data, the red dashed line represents the reconstructed data obtained using the reconstruction algorithm in one direction, and the green solid line represents the difference between the two. The difference between the reconstructed data and the test data is only approximately constant rather than a linear function when the test data is an axisymmetric function. From a one-dimensional perspective, our primary focus is on the variation trend of the reconstructed data, and any constant difference introduced by the reconstruction can be neglected.

**Figure 4:**
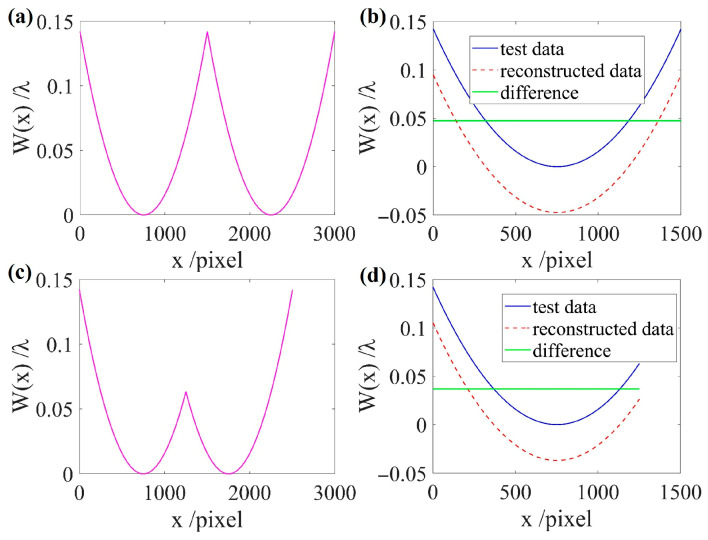
Comparison of reconstruction results after even prolongation of the functions. (**a**) An axisymmetric function undergoes even prolongation; (**b**) reconstruction result of (**a**); (**c**) a non-axisymmetric function undergoes even prolongation; (**d**) reconstruction result of (**c**).

However, the test data is not necessarily an axisymmetric function. The reconstruction results after even prolongation for the two types of functions in Figure 3 are shown in Figure 4.

The pink curves in Figure 4a and Figure 4c represent the even prolongation of the test data shown in Figure 3a and Figure 3b, respectively. In Figure 4b,d, the blue solid line represents the known test data, the red dashed line represents the reconstructed data obtained using the reconstruction algorithm in one direction, and the green solid line represents the difference between the two sets of data. The PV values of the two sets of difference data are approximately $6.7\times 10^{-12}$λ and $1.1\times 10^{-11}$λ, respectively. Therefore, regardless of the function type, the difference between the evenly prolonged test data and the reconstructed data can be considered a constant. The constant error in the reconstructed data originates from the fact that the one-direction reconstruction algorithm performs integration only along the x direction; therefore, the constant error is introduced in the y direction. In this section, we only focus on the information variation in the x direction, so the constant error in the y direction can generally be ignored and suppressed using the noise suppression method. This demonstrates that even prolongation is crucial for achieving complete reconstruction of wavefront data. By incorporating wavefront even prolongation into the reconstruction algorithm in one direction, the wavefront information is preserved, improving the reconstruction accuracy.

### 3.3. Noise Suppression Method

In practice, the method described in Equation (8) suffers from division by a small quantity, and division by *A*(*u*) can also cause an amplification of noise, thus affecting the reconstruction result. The solution to this is to add a spectral signal-to-noise ratio distribution term *SN*(*u*, *v*) in the denominator.

(14) $$G(u,v)=\frac{A{\left(u\right)}^{*}}{{\left|A(u)\right|}^{2}+SN(u,v)}W(u,v),$$

where *SN*(*u*, *v*) = *u*^2^ + β*v*^2^, which can be expressed using different mathematical formulations depending on the chosen model. β denotes the smoothing factor.

Noise propagates during reconstruction in one direction, resulting in stripe artifacts. *SN*(*u*, *v*) can play a role in noise suppression, where the value of β determines the effectiveness of noise suppression. The physical meaning of β lies in only smoothing the y direction during integration along the x direction by multiplying β by *v*^2^ to suppress constant error in the y direction and noise [38].

**Figure 5:**
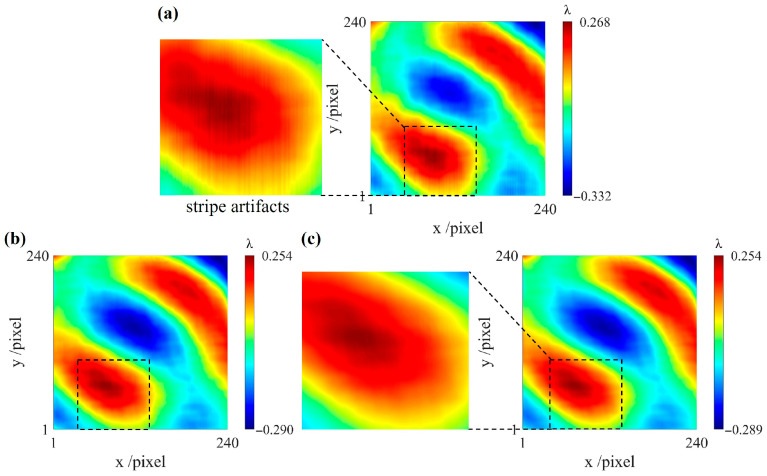
Comparison of noise suppression effects in one-direction reconstruction. (**a**) β = 0; (**b**) β = 0.01; (**c**) β = 0.1.

Figure 5a shows that noise produces obvious stripe artifacts in the reconstructed result when β = 0. To better compare the smoothing effect, the same regions in Figure 5a–c are outlined with black dashed lines. As β increases, the stripe artifacts gradually disappear, and the reconstructed result becomes smoother. In Figure 5b, the stripe artifacts disappear when β = 0.01, whereas increasing β further may cause partial distortion.

### 3.4. Simulation Experiment

**Figure 6:**
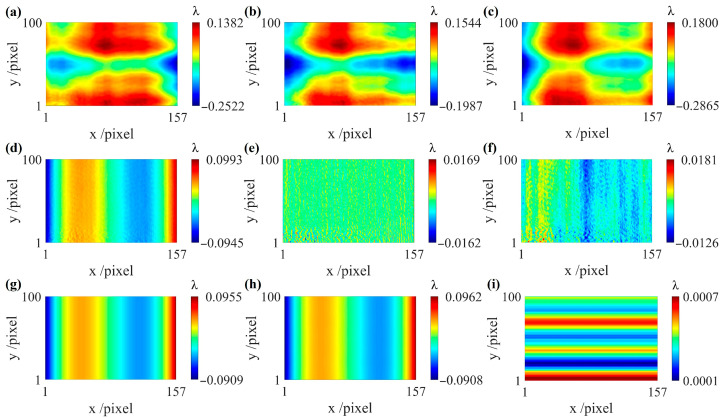
Simulation results of the reconstruction algorithm in one direction. (**a**) Wavefront error *W_x_*_1_; (**b**) wavefront error *W_x_*_2_; (**c**) wavefront error *W_x_*_3_; (**d**) first-order differential wavefront *g_x_*_1_; (**e**) second-order differential wavefront *g_xx_*; (**f**) reconstruction results of (**e**) in one direction; (**g**) simulated misalignment aberrations $\phi_{MAx}$; (**h**) reconstructed misalignment aberrations $\phi_{MAx}\prime$; (**i**) difference map of (**h**) and (**g**).

Simulation experiments based on the reconstruction algorithm in one direction are performed, with the CSCP being translated only along the x direction. The CSCP was assumed to have a size of 157 × 100 pixels, the interferometer wavelength is 632.8 nm, and the shear amount is 1 pixel. The simulation results are shown in Figure 6.

Figure 6a–c show the three sets of wavefront errors, *W_x_*_1_, *W_x_*_2_, and *W_x_*_3_, which are conjugately translated along the x direction. Figure 6g shows the $\phi_{MAx}$ generated by simulation. The reconstructed $\phi_{MAx}\prime$ is obtained using the proposed algorithm, as shown in Figure 6h. From Figure 6g,h, we can clearly see that the two distributions are highly similar, as the PV and RMS values of their difference map are 0.0006λ and 0.0004λ, thus verifying the feasibility of the proposed method. Based on the discussions in Section 3, the reconstruction algorithm in one direction can accurately reconstruct the misalignment aberrations, yielding the first-order x-differential wavefront that is free of misalignment aberrations. Combined with the directly measured first-order y-differential wavefront, the cylindrical surface figure can be precisely recovered.

## 4. Experimental Results

**Figure 7:**
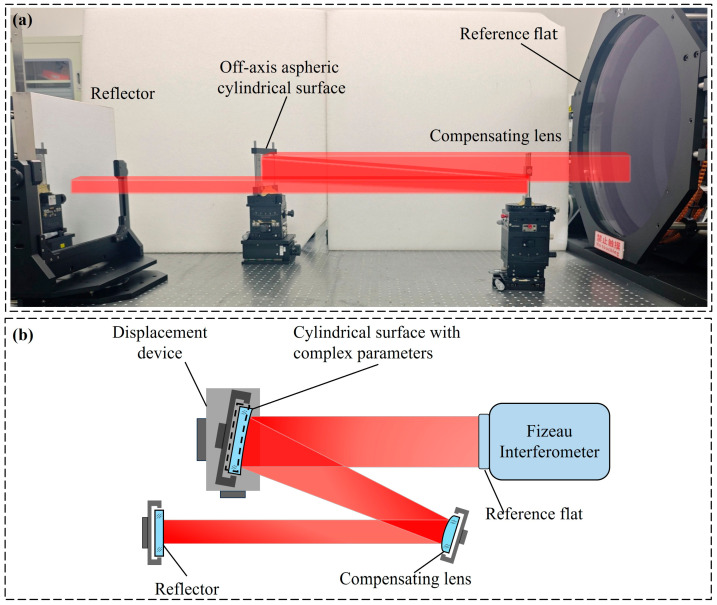
Experimental layout for measuring the test cylindrical surface. (**a**) Experimental setup; (**b**) graphical scheme (top view) of the experimental setup.

To verify the effectiveness of the proposed method, experiments were carried out using a Fizeau interferometer based on the non-null interferometric system described in Section 2. The experimental layout shown in Figure 7a was built to measure the test cylindrical surface. The Fizeau interferometer employs a wavelength of 632.8 nm, with an aperture diameter of 600 mm. The CCD provides a maximum resolution of 3092 pixels × 3092 pixels. The off-axis distance of the test cylindrical surface is 422.054 mm, the conic constant is −0.674, and the curvature radius is 2289.1 mm. The compensating lens has a conic constant of 0 and a curvature radius of 790.23 mm, with PV and RMS values of 6.48 *λ* and 1.26 *λ*, respectively. The compensating lens only compensates for part of the wavefront aberrations.

**Figure 8:**
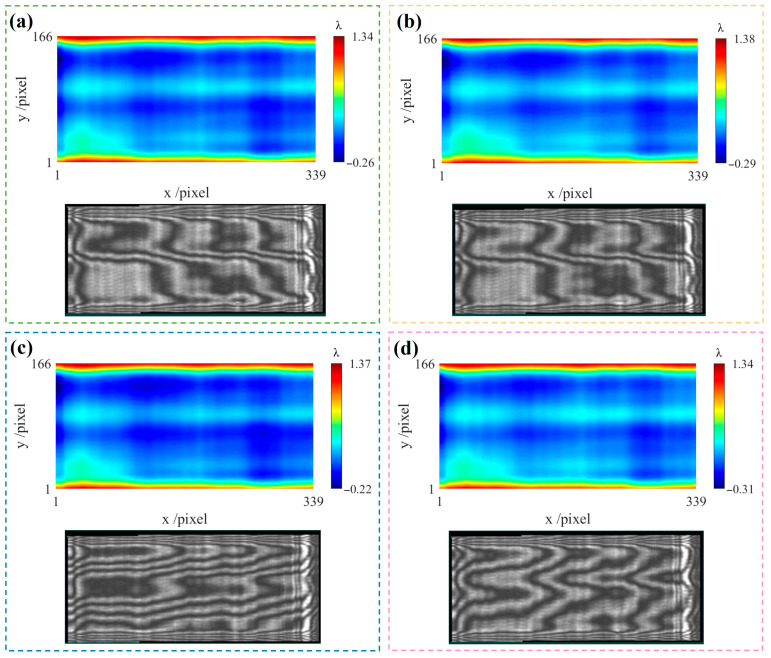
Interference fringes and corresponding measurement results at different translation positions. (**a**) Measurement result at reference position *W_x_*_2_; (**b**) measurement result after translation along the y-axis direction, *W_y_*_1_; (**c**) measurement result after translation along the positive x-axis direction, *W_x_*_1_; (**d**) measurement result after translation along the negative x-axis direction, *W_x_*_3_.

The displacement device with six degrees of freedom is used to translate the test cylindrical surface along orthogonal directions, enabling the shearing process. The displacement resolution is 1 µm along the x-axis and 5 µm along the y-axis. The measurement results and interference fringes are shown in Figure 8a–d. During the actual measurement process, the pose of the compensating lens was adjusted using the displacement device to make the interference fringes as sparse as possible, thereby obtaining the measurement result at the reference position, as shown in Figure 8a. The test cylindrical surface was first translated along the y direction, and the corresponding measurement result is shown in Figure 8b. It can be seen from the interference fringes that translating along the y direction does not introduce misalignment aberrations. Subsequently, the test cylindrical surface was translated back to the reference position. Two sets of measurement results, obtained through conjugate translations along the x direction, are shown in Figure 8c,d. The data were directly measured after translation, without pose adjustment or aberration removal based on existing models.

**Figure 9:**
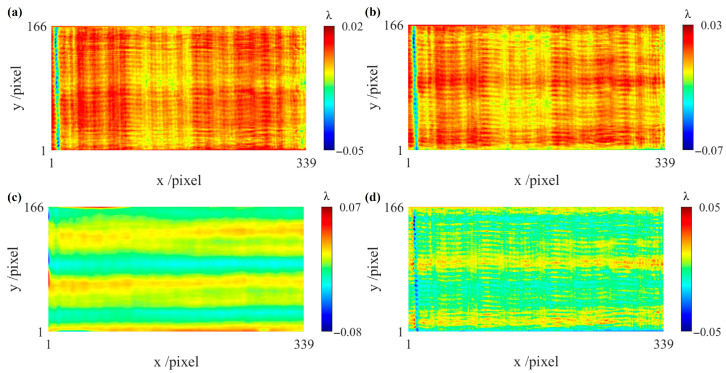
Differential wavefronts. (**a**) First-order x-differential wavefront *g_x_*_1_; (**b**) first-order x-differential wavefront *g_x_*_2_; (**c**) first-order y-differential wavefront *g_y_*; (**d**) second-order x-differential wavefront *g_xx_*.

Figure 9a,b show the two first-order x-differential wavefronts, both exhibiting misalignment aberrations. Figure 9c shows the first-order y-differential wavefront. Figure 9d shows the second-order x-differential wavefront, indicating that subtracting Figure 9a from Figure 9b effectively eliminates the misalignment aberrations.

**Figure 10:**
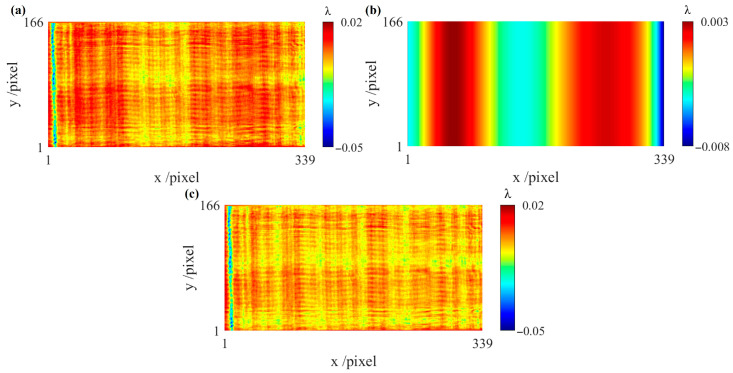
Results of the reconstruction algorithm in one direction and comparative analysis. (**a**) The measured first-order differential wavefront *g_x_*_1_; (**b**) misalignment aberrations $\phi_{MAx}$; (**c**) the reconstructed first-order differential wavefront obtained by subtracting (**b**) from (**a**).

Based on the differential wavefronts in Figure 9, the feasibility of the reconstruction algorithm in one direction in actual experiments can be verified. The results are shown in Figure 10a–c. Figure 10a shows the measured first-order differential wavefront *g_x_*_1_. Figure 10b shows the $\phi_{MAx}$ obtained through the proposed algorithm. Figure 10c shows the reconstructed first-order differential wavefront *g_x_*_1_. A comparison between Figure 10a,c indicates that the overall variation trends of the two are generally consistent, and the misalignment aberrations are removed in Figure 10c.

**Figure 11:**
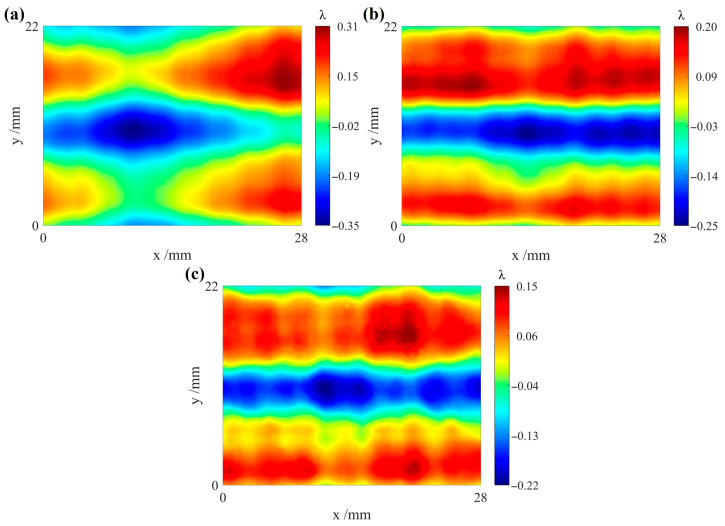
Comparison of measurement results between the proposed method and CGH measurements. (**a**) Reconstructed result of test cylindrical surface without removal of misalignment aberrations in the differential wavefront; (**b**) reconstructed result of test cylindrical surface obtained using the proposed algorithm; (**c**) measurement result by CGH.

Using the first-order differential wavefronts in the orthogonal directions with misalignment aberrations removed, the surface figure of the test cylindrical surface can be reconstructed via the Hudgin model method. To verify the accuracy of the reconstruction results by our method, we used a CGH to measure the test cylindrical surface and compared the results with those of the proposed algorithm in the same region. Since the edge regions of the differential wavefronts can affect the reconstruction results, the central region is selected for comparison. The reconstructed results of the test cylindrical surface without and with the removal of misalignment aberrations of the differential wavefronts are shown in Figure 11a,b, respectively, showing significant differences. Figure 11c shows the measurement result using a CGH. The natural distortion in the CGH detection has already been corrected [39]. The distributions in Figure 11a,c are quite different. Figure 11a clearly exhibits misalignment aberrations, with a PV value of 0.66*λ* and an RMS value of 0.14*λ*. From Figure 11b,c, it is evident that the testing result obtained by our method agrees well in distribution with the result obtained from the CGH test. The PV and RMS values of Figure 11b are 0.45*λ* and 0.12*λ*, respectively, compared with 0.37*λ* and 0.09*λ* for Figure 11c. The corresponding differences in PV and RMS values are 0.08*λ* and 0.03*λ*, respectively. These findings suggest that the proposed method is effective in removing misalignment aberrations from the measured data.

**Figure 12:**
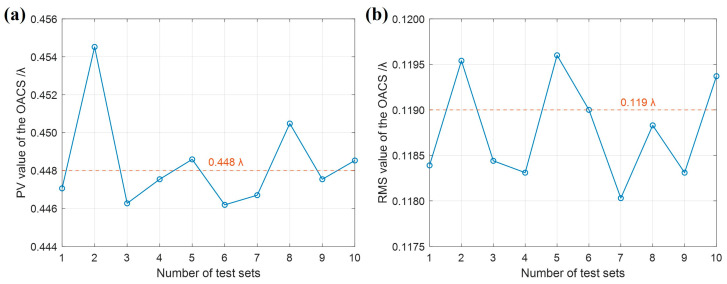
Reproducibility test. (**a**) PV values of the reconstruction results from 10 sets of repeated tests; (**b**) RMS values of the reconstruction results from 10 sets of repeated tests.

To further verify the repeatability and accuracy of the proposed method, ten sets of tests were performed, and the reconstructed results of test cylindrical surface were statistically analyzed, as shown in Figure 12a,b. The average value and standard deviation of PV values of the reconstructed results from ten sets of tests are 0.448*λ* and 0.003*λ*, respectively. The average value and standard deviation of RMS values are 0.119*λ* and 0.001*λ*, respectively, with the repeatability being better than *λ*/1000. These results further demonstrate the reliability and accuracy of the proposed method for the test cylindrical surface introduced in this paper.

## 5. Discussion

The method proposed in this paper can effectively reconstruct and eliminate misalignment aberrations in differential wavefronts and reconstruct the surface figure of a test cylindrical surface. However, compared with the CGH measurement results, a slight discrepancy in reconstruction accuracy still exists. This discrepancy is mainly caused by various errors, and the detailed analysis and corresponding optimization methods are as follows:

(a) When the CSCP is conjugately translated along the x direction, inherent positioning errors of the displacement device, reading deviations introduced by manual operation, tilt deviations, and non-ideal repeatability are difficult to completely avoid. If the actual displacement distances of the two times are inconsistent with the theoretical shear amount, the misalignment aberrations cannot be fully eliminated in the second-order differential wavefront, thereby degrading the reconstruction accuracy. To address this issue, a computer-controlled high-precision displacement device can be employed, ensuring that the shear amount maintains an integer multiple relationship with the displacement resolution, so as to avoid errors caused by displacement mismatch. According to the references, the measurement error of two-dimensional pseudo lateral shearing interferometry is the smallest when the shear amount is one pixel [31,32].
(b) The second-order differencing in the algorithm essentially approximates continuous derivatives using discrete data, and the finite sampling resolution introduces approximation errors that are further amplified in the second-order differencing. Simultaneously, second-order differencing is equivalent to a high-pass filter in the frequency domain, significantly amplifying high-frequency noise. Although a smoothing factor β has been introduced in this study to suppress noise, its optimal value and the specific form of the regularization term (the spectral signal-to-noise ratio distribution) still warrant further investigation. In the future, increasing the sampling density and optimizing the regularization strategy could achieve a better balance between noise suppression and signal fidelity.
(c) The non-null interferometric system used in this study has a relatively long interferometric cavity, making the measurements sensitive to environmental disturbances. During the acquisition of the three wavefronts along the x direction, minor vibrations or airflow variations can disrupt the phase relationship among *W_x_*_1_, *W_x_*_2_, and *W_x_*_3_, introducing random phase noise into the differential wavefronts. This issue can be mitigated by improving the stability of the experimental environment and by performing multiple acquisitions under the same conditions and averaging the results.
(d) The experiments were conducted on a single off-axis aspheric cylindrical surface, and a limited number of repeatability tests were performed. Therefore, the present study is subject to the limitation of single-sample validation and cannot fully represent the measurement performance of the proposed method under different parameter conditions. However, two-dimensional pseudo lateral shearing interferometry is a generalized interferometric measurement method and is theoretically applicable to CSCPs with different parameter configurations. Considering the applicable parameter ranges of CSCPs described in Section 2, compensating lenses for CSCPs with different parameter configurations can be designed through optical simulations based on the non-null interferometric system shown in Figure 7b, enabling non-null testing of various CSCPs. Therefore, the proposed method can be extended to different CSCP configurations and achieve similar measurement performance. In addition, the size of the compensating lens determines the measurable range of the CSCP, and a series of compensating lenses will be designed in future work to continuously improve the universality of the system.

In summary, the feasibility of the proposed method has been verified using a laboratory-built measurement system. With systematic optimization in the aspects discussed above, the measurement accuracy of the method can be further improved, thereby promoting its application in practical industrial inspections.

## 6. Conclusions

In this paper, we introduce a novel non-null interferometry method for CSCPs. The method is based on small translations of the CSCP in orthogonal directions, combined with an additional conjugate translation along the x direction. Misalignment aberrations that have been introduced during the translation are effectively eliminated through second-order differencing in the x direction. For the single-degree-of-freedom motion along the x direction, the reconstruction algorithm in one direction is further proposed. By only using the partial derivative in the x direction, the wavefront error of misalignment aberrations can be reconstructed, enabling high-precision recovery of the CSCP surface. The advantage of the proposed method lies in directly processing the measurement data without the need for complex matrix operations or model matching. The experimental results demonstrate that the proposed method exhibits good repeatability, with a precision better than *λ*/1000 RMS. The reconstructed CSCP surface figure achieves PV and RMS values of 0.45*λ* and 0.12*λ*, respectively, close to the 0.37*λ* PV and 0.09*λ* RMS obtained via CGH null testing, indicating similar accuracy. Furthermore, we also analyze various error sources and propose subsequent solutions.

Compared with null interferometric testing, the proposed method has greater versatility, and the reconstructed results are in good agreement with those obtained from CGH measurements, providing a feasible solution for CSCP testing. In addition, since translating the CSCP along the y direction does not introduce misalignment aberrations, future research will explore a surface reconstruction method based solely on a single translation along the y direction, further simplifying the experimental procedure and algorithm structure, and thereby improving testing efficiency.

## Data Availability

The data underlying the results presented in this paper are not publicly available at this time but may be obtained from the authors upon reasonable request.
